# Neural and psychological mechanisms underlying compulsive drug seeking habits and drug memories – indications for novel treatments of addiction*

**DOI:** 10.1111/ejn.12644

**Published:** 2014-06-17

**Authors:** Barry J Everitt

**Affiliations:** Department of Psychology, Behavioural and Clinical Neuroscience Institute, University of CambridgeDowning Street, Cambridge, CB2 3EB, UK

**Keywords:** cocaine, compulsion, habits, reconsolidation, relapse, striatum

## Abstract

This review discusses the evidence for the hypothesis that the development of drug addiction can be understood in terms of interactions between Pavlovian and instrumental learning and memory mechanisms in the brain that underlie the seeking and taking of drugs. It is argued that these behaviours initially are goal-directed, but increasingly become elicited as stimulus–response habits by drug-associated conditioned stimuli that are established by Pavlovian conditioning. It is further argued that compulsive drug use emerges as the result of a loss of prefrontal cortical inhibitory control over drug seeking habits. Data are reviewed that indicate these transitions from use to abuse to addiction depend upon shifts from ventral to dorsal striatal control over behaviour, mediated in part by serial connectivity between the striatum and midbrain dopamine systems. Only some individuals lose control over their drug use, and the importance of behavioural impulsivity as a vulnerability trait predicting stimulant abuse and addiction in animals and humans, together with consideration of an emerging neuroendophenotype for addiction are discussed. Finally, the potential for developing treatments for addiction is considered in light of the neuropsychological advances that are reviewed, including the possibility of targeting drug memory reconsolidation and extinction to reduce Pavlovian influences on drug seeking as a means of promoting abstinence and preventing relapse.

## Introduction

Many people take drugs that are addictive and do so for different reasons, in different ways and in different contexts, for example the social drinking of alcohol, which is acceptable in some but not all societies, or the more solitary intravenous use of drugs such as heroin. These drugs are powerfully reinforcing and may cause intense subjective effects that, once experienced, lead to further experimentation and drug taking experiences. While such drug taking may continue on an occasional basis for a long period of time, some individuals lose control over their drug use and are unable to stop. They compulsively seek and take drugs despite the obviously deleterious effects of doing so on their personal and social wellbeing, and often in the face of danger and punishment by the legal system (American Psychiatric Association, 2013). It is therefore important to disentangle the mechanisms that underlie drug use from those whereby vulnerable individuals become addicted. Understanding the neural and psychological basis of the transition from initial drug use to compulsive use when addicted or dependent has been the focus of remarkably successful experimental research in animals and, increasingly, in humans for nearly half a century. Consequently, we now have a wealth of data on the initial molecular actions of addictive drugs, where in discrete brain regions they produce their reinforcing effects and how repeated or chronic drug taking changes the brain, thereby contributing to the emergence of compulsive drug use (Koob & Le Moal, 2005a; Nestler, 2005; Russo *et al*., 2010; Nestler, 2014). The factors that might predispose individuals to lose control over drug use are being defined both in animals (Dalley *et al*., 2007; Belin *et al*., 2008; Dilleen *et al*., 2012) and, through the study of addicted individuals and their siblings, also in humans, leading to the identification of endophenotypes relevant to drug addiction and related neuropsychiatric disorders (Ersche *et al*., 2010). Despite this very considerable body of knowledge, there has been disappointingly little progress in bringing novel and effective treatments to the clinic, even though there is a major unmet need and a plethora of viable targets – a situation that it is hoped may change in the near future.

This review is not intended to be exhaustive, but to reflect my FENS-EJN award lecture delivered at the FENS Forum in Barcelona in 2012, which summarized aspects of the theoretical basis and results of some of our research. One of the starting points of this research was that the investigation of the molecular and neurochemical correlates of the reinforcing effects of acute drug self-administration and of chronic drug use will be relevant to understanding addictive behaviour only provided it is embedded in a more integrative approach allowing for their interpretation in behavioural and cognitive terms (Everitt *et al*., 2001). Thus, while it had long been accepted that drugs have reinforcing effects, generally assumed to depend upon increased dopamine (DA) transmission in the nucleus accumbens (NAcb) and associated ventral striatal areas (Wise, 2008) (although whether this explains all reinforcing effects of all classes of abused drugs remains contentious), it was also clear that responses to drugs can acquire motivational significance by being associated with environmental stimuli through pavlovian conditioning (Gawin & Kleber, 1986; O'Brien *et al*., 1998). These drug-associated conditioned stimuli (CSs) may then predict drug availability, evoke memories of a drug's effects, or of withdrawal, to result in craving even long into abstinence, and, perhaps most importantly, may elicit and maintain the instrumental behaviours of drug seeking and taking (Wikler, 1965; Grant *et al*., 1996; Childress *et al*., 1999; Garavan *et al*., 2000; Robbins & Everitt, 2002). This led us to suggest that the complex processes underlying addiction can be understood in terms of the operation of the brain's pavlovian and instrumental learning and memory systems, and their subversion by the potent actions of self-administered addictive drugs on dopaminergic transmission within corticostriatal systems that normally mediate learning and memory processes in the context of natural rewards (Robbins & Everitt, 1999; Everitt & Robbins, 2005; Belin *et al*., 2013).

**FIG. 1:**
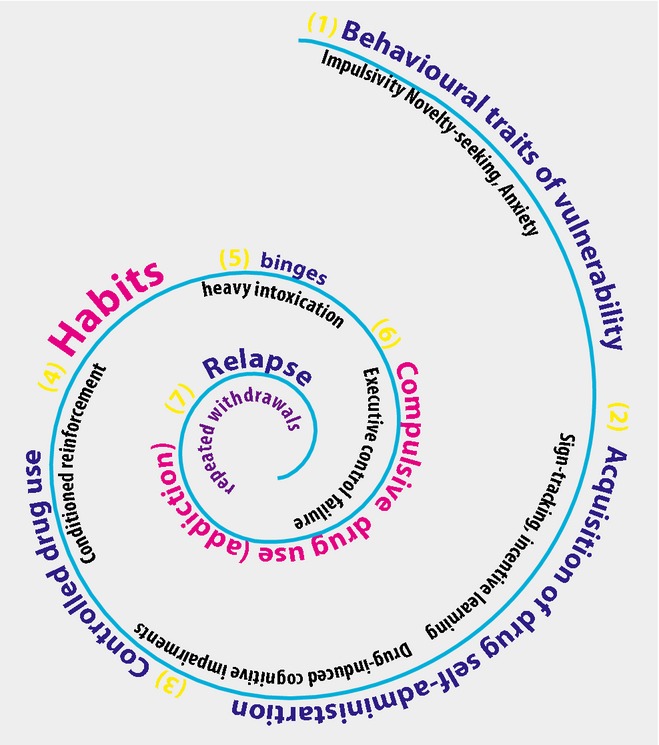
A schematic summary of the psychological processes that may underlie the transition from voluntary drug seeking, through loss of control over drug use to the emergence of compulsive drug seeking habits. Although depicted as a spiral (see Koob & Le Moal, 2001), these processes may also occur in parallel, as discussed in the text. The notion of vulnerability is also captured in the spiral, determining that some but not all individuals ultimately seek and take drugs compulsively, in part through the loss of inhibitory control over drug seeking habits. (Figure generously provided by David Belin, 2014).

Our proposition was – indeed is – that drug addiction can be viewed as the endpoint of a series of transitions from initial voluntary, or recreational, drug taking through progressive loss of control over drug use (Robbins & Everitt, 1999; Everitt & Robbins, 2005; Belin *et al*., 2013). Drug seeking thereby becomes increasingly elicited by and under the control of the drug-associated stimuli established through pavlovian association with early and repeated drug effects, so that it is eventually consolidated as a maladaptive stimulus–response (S-R) habit. Ultimately drug seeking and taking become compulsive in individuals vulnerable to addiction (Fig. 1).

**FIG. 2:**
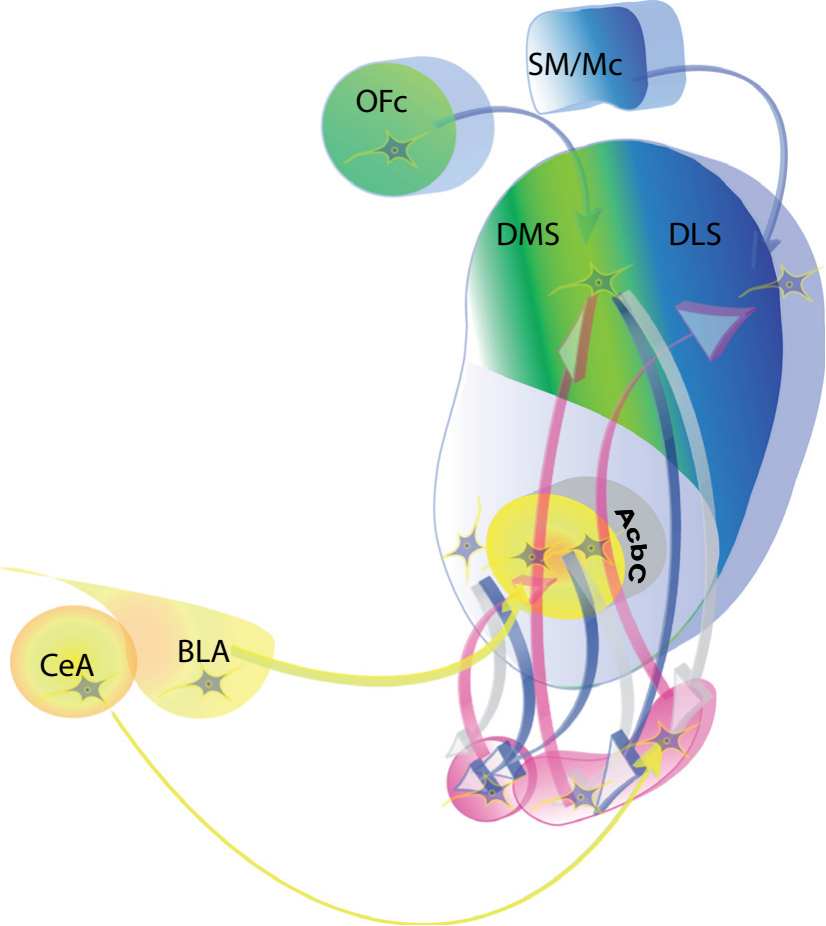
The neural circuitry of goal-directed (controlled by Action-Outcome associations) and habitual (controlled by stimulus–response associations) drug seeking behaviour. Basolateral amygdala (BLA) – nucleus accumbens core (AcbC) circuitry (yellow) mediates the impact of conditioned reinforcement on drug seeking behaviour, modulated by the actions of the mesolimbic DA system originating in the midbrain (pink). The dorsomedial striatum (DMS) with its orbitofrontal cortex (OFc) inputs (green), together with medial prefrontal cortex (not illustrated) mediate the acquisition and performance of the goal-directed seeking of drugs (cocaine) and ingestive rewards. The dorsolateral striatum (DLS) and its sensorimotor cortical afferents (SM/Mc) mediate the acquisition and performance of well-established drug seeking habits and stimulus–response control. Also illustrated is the serial connectivity linking the nucleus accumbens with the DMS and DLS via recurrent connections with the midbrain DA neurons (pink – interconnecting pink and blue arrows, after Haber *et al*., 2000) mediating intrastriatal shifts in the control over drug seeking from ventral to dorsal striatum. Substantia nigra neurons projecting to the DLS are also regulated by afferents from the central nucleus of the amygdala (CeN). (Figure generously provided by David Belin, 2014).

The notion of progression from use to abuse to addiction features in diagnostic, clinical and experimental accounts of addiction (Robinson & Berridge, 1993; O'Brien & McLellan, 1996; Koob & Le Moal, 2001; Everitt & Robbins, 2005; American Psychiatric Association, 2013; Belin *et al*., 2013). Our experimental approach involved bringing a learning theory analysis of pavlovian and instrumental learning processes, and interactions between them, to the investigation of the neural mechanisms underlying both drug seeking and taking (Everitt *et al*., 2001). We hypothesized that the transition from voluntary to habitual drug seeking may reflect a devolution in behavioural control from one corticostriatal system to another, more specifically from ventral to dorsal striatal systems, mediated by the recruitment by drugs of the serial interconnectivity of striatal regions with midbrain DA neurons (Fig. 2). This transition may facilitate the emergence of compulsivity, resulting from a progressive decrease in inhibitory control by prefrontal cortical areas over drug seeking habits (Everitt & Robbins, 2005).

Pavlovian mechanisms influencing drug self-administration are a feature of several major theories of addiction, whether based on incentive or opponent processes (Wikler, 1965; Stewart *et al*., 1984; Robinson & Berridge, 1993; Schulteis *et al*., 2000; Everitt *et al*., 2001). However, it seemed both timely and important to focus on the obvious fact that drug seeking and taking are instrumental behaviours and that the general concept of positive reinforcement conflates at least two different processes which have been identified by contemporary analyses of instrumental conditioning with conventional reinforcers (Dickinson, 1985; Dickinson & Balleine, 1994). The first is a declarative associative process based upon knowledge of the relationship between instrumental behaviour, or action (A) and its outcome (O), taking the form of intentional, goal-directed actions. If the outcome is ‘devalued’, goal-directed actions are greatly decreased. The second is an S-R mechanism by which reinforcers strengthen an association between the response and the contextual and discrete stimuli present at the time of reinforcement. Habitual behaviour controlled by this process is elicited automatically by CSs, rather than the representation of the goal, as shown by the fact that if the outcome is devalued, seeking behaviour persists.

In studying these processes in animals self-administering cocaine, heroin and more recently alcohol, we felt it important to distinguish between ‘drug seeking’ and ‘drug taking’ behaviour, not least because it is well established that appetitive behaviour (seeking) is readily dissociated from consummatory behaviour (taking) in studies of ingestive and sexual behaviour (Blackburn *et al*., 1987, 1989; Cador *et al*., 1989; Everitt, 1990). Drug taking is most frequently studied and involves the reinforcement of simple lever presses [usually under continuous reinforcement whereby every lever press is followed by an i.v. drug infusion – fixed ratio (FR) 1]. It is this methodology, developed by Weeks (1962), that has been used to define the nucleus accumbens and adjacent ventral striatal structures as a key site for the reinforcing effects of stimulants, opioids and other drugs of abuse (Roberts & Koob, 1982; Wise, 2004; Ikemoto *et al*., 2005). But there is a much less tight and predictable relationship between response and outcome when individuals seek drugs in more complex, or real world environments, where reinforcement is often delayed and drug seeking must be maintained over long periods of time (Everitt & Robbins, 2005). Drug seeking is also markedly influenced by drug-associated CSs that in humans induce subjective craving states, drug seeking and relapse after abstinence (Grant *et al*., 1996; Childress *et al*., 1999; Garavan *et al*., 2000).

We invested considerable effort in establishing drug seeking under a second-order schedule of reinforcement as it captures delays to reinforcement (15–60 min), critical dependence on the contingent presentation of cocaine-associated CSs acting as conditioned reinforcers, as well, of course, as the delivery of drug (cocaine, heroin and more recently alcohol) (Arroyo *et al*., 1998; Everitt & Robbins, 2000). It can be studied in acquisition, or after a change in response contingency requirements when under A-O control (Murray *et al*., 2012a). Drug seeking can also be studied, at the psychological (CS omission) and neurobiological level, when well established or habitual and under S-R control after weeks of training, as well as at time points between these two extremes (Murray *et al*., 2012a). Using this behavioural approach, we have demonstrated the engagement of different territories of the striatum during the transition from goal-directed to habitual drug seeking. We also developed a second task, described as a seeking–taking chained schedule of drug reinforcement in which seeking responses are distinct and separate from taking (consummatory) responses and can be manipulated independently, thereby allowing the associative structure, A-O or S-R, underlying seeking to be investigated (Olmstead *et al*., 2000, 2001). This task has also been employed to study drug seeking when it is risky because of the threat or actual occurrence of unpredictable and intermittent punishment, enabling us to model compulsive drug seeking, defined as persisting in the face of adverse consequences (Vanderschuren & Everitt, 2004; Pelloux *et al*., 2007).

## Acquisition and performance of drug seeking – pavlovian and instrumental processes

There is now abundant evidence showing that limbic cortical–ventral striatal circuitry mediates pavlovian influences on appetitive behaviour, including drug seeking (Everitt *et al*., 1999; Robbins *et al*., 2008). The basolateral amygdala (BLA) is a major source of afferents to the ventral striatum, especially to the nucleus accumbens core, but also to the shell region (Wright *et al*., 1996). This system provides the basis for interactions between stimulus–reward associative processing in the amygdala and the primary site mediating the reinforcing effects of addictive drugs, the ventral striatum, thereby underlying both pavlovian and instrumental appetitive behaviours – much as Mogenson *et al*. (1984) had predicted when describing this system as a ‘limbic-motor interface’ (Fig. 2). Selective BLA lesions or NAcb core lesions markedly impaired the acquisition of cocaine seeking under a second-order schedule (Whitelaw *et al*., 1996; Ito *et al*., 2004), as did a neurochemical functional disconnection of this circuitry by combining unilateral manipulations of each structure in opposite hemispheres (Di Ciano & Everitt, 2004). Lesions to the BLA and NAcb core also impaired the acquisition of heroin seeking (Alderson *et al*., 2001). Note that neither BLA nor NAcb core lesions (nor NAcb shell lesions) impaired cocaine (or heroin) self-administration, i.e. drug taking. Lesion-induced deficits were only seen when seeking depended on the presentation of cocaine-associated conditioned reinforcers and when the drug infusions were delayed. The mechanisms underlying drug taking and drug seeking under the influence of pavlovian drug-associated stimuli are therefore dissociable. Moreover, the neural basis of the A-O learning processes supporting instrumental drug taking and seeking must lie at least in part in other loci (see below).

The effects of lesions of the BLA or NAcb core are consistent with their established, interacting involvement in conditioned reinforcement (Cador *et al*., 1989; Robbins *et al*., 1989). The amygdala–NAcb system also mediates pavlovian approach behaviour (Parkinson *et al*., 1999, 2000; Flagel *et al*., 2011) and pavlovian-instrumental transfer, or PIT (Hall *et al*., 2001; Corbit & Balleine, 2005). Perhaps less widely appreciated is the importance of both the BLA and the NAcb core in mediating delays to reinforcement, a process that is markedly facilitated by the presentation of conditioned reinforcers (Cardinal *et al*., 2004; Winstanley *et al*., 2004). Both BLA and NAcb core lesions resulted in impulsive behaviour, with animals choosing small, immediate over larger, delayed rewards. The NAcb core is further required for instrumental learning when there is a delay between response and reinforcer (Cardinal & Cheung, 2005), again probably because it mediates CSs presented during the delay acting to reinforce and support instrumental responding that leads ultimately to reward (Cardinal *et al*., 2002).

Relapse to drug seeking after instrumental extinction, which also depends on drug CSs acting as conditioned reinforcers, as well as the strengthening of the ability of conditioned reinforcing effects of drug-associated CSs during abstinence to precipitate relapse (‘incubation’; Grimm *et al*., 2001; Lu *et al*., 2004), are mediated by processing in the amygdala and NAcb and interactions between them, further supporting the importance of this circuitry in drug seeking [reviewed extensively (McFarland & Kalivas, 2001; See *et al*., 2003; Lu *et al*., 2005)].

The importance of the ventral striatum, including the NAcb, in mediating drug reinforcement, and of the amygdala–NAcb circuitry in mediating conditioned reinforcement, PIT and pavlovian approach (reviewed by Cardinal *et al*., 2002; Cardinal & Everitt, 2004) is well established. However, the striatal (and cortical) mechanisms underlying the acquisition of drug seeking when goal-directed and controlled by instrumental A-O processes has only recently been revealed, being informed by the investigation of responding for ingestive reinforcers. Here, the posterior dorsomedial striatum (pDMS) has been shown to be of key importance. Using the procedure of outcome devaluation to probe the A-O vs. S-R control over responding for food, lesions, *N*-methyl-d-aspartate (NMDA) receptor blockade or disruption of extracellular signal-regulated kinase (ERK) in the pDMS prevented A-O learning during the acquisition of food-reinforced responding (Yin *et al*., 2005a,b; Shiflett *et al*., 2010). This does not mean that animals do not respond for food. Instead these manipulations result in a loss of sensitivity to outcome devaluation; the animals still eat and food retains its reinforcing properties (which are correlated with DA release in the NAcb; Blackburn *et al*., 1986; Bassareo & Di Chiara, 1999).

But the neural parsing of reward or reinforcement, pavlovian influences on instrumental behaviour and A-O instrumental learning itself is difficult, because these processes are closely intertwined. However, we have shown that DA receptor blockade in the pDMS dose-dependently impaired the acquisition of cocaine seeking when animals had to adapt to a change in contingency in responding for both the CS and the cocaine when under A-O control. The same manipulation had no effect after extended, or over-, training, suggesting the neural locus of control over behaviour shifts with experience (Murray *et al*., 2012a). Nor could the effects of pDMS DA receptor blockade on learning and goal-directed performance be attributed to changes in cocaine reinforcement, as the effects on seeking acquisition were measured before any influence of self-administered cocaine infusion in each seeking test session, and also because pDMS DA receptor blockade had no effect on cocaine taking (i.e. simple self-administration of the drug; Murray *et al*., 2012a). Interestingly, selective lesions of the orbital prefrontal cortex (OFC) also impaired the acquisition of cocaine seeking (Everitt *et al*., 2007). The OFC projects richly to the pDMS and interactions between these structures have been implicated in goal-directed instrumental action (Schoenbaum & Roesch, 2005; Ostlund & Balleine, 2007). In addition the OFC projects reciprocally to the BLA and this system plays a role in conditioned reinforcement processes (Parkinson *et al*., 2001; Burke *et al*., 2008). Taken together, then, these data argue for the parallel involvement of the BLA, NAcb core, pDMS and OFC in the acquisition and performance of goal-directed cocaine seeking maintained over delays to drug reward and sustained by drug-associated conditioned reinforcement (Fig. 2).

## The emergence of drug seeking habits and dorsolateral striatal control

After extended training under a second-order schedule of reinforcement for cocaine, DA receptor blockade in the pDMS or NAcb no longer impaired drug seeking. Instead, the same manipulation in the anterior dosolateral striatum (aDLS) did – yet this had no effect at earlier stages when seeking behaviour was sensitive to pDMS DA receptor blockade (Murray *et al*., 2012a). This shift to aDLS control (Fig. 2) was predicted by the earlier finding that extracellular DA was increased in the DLS, but not NAcb core or shell, during a 1-h period of cocaine seeking in rats that had been trained extensively over a period of 2 months under the same schedule of reinforcement (Ito *et al*., 2002). We interpreted these results on the backdrop of an earlier literature on the role of the dorsal striatum in habit learning (Mishkin *et al*., 1984) and also a rapidly growing body of data on food-reinforced instrumental behaviour, where insensitivity to reinforcer devaluation had revealed that the aDLS is involved specifically in S-R learning and performance (Yin *et al*., 2004, 2006). An interval-based second-order schedule such as the one adopted in our experiments, where there is a weaker relationship between response and outcome compared with ratio, especially low ratio, reinforcement schedules, is much more likely to result in S-R, or habit-based, responding (Dickinson *et al*., 1995). But devaluing cocaine by specific satiety, lithium chloride (LiCl)-induced malaise, or even contingency degradation has proven to be extremely difficult and not very effective when an intravenously self-administered drug like cocaine is the reinforcer. The reasons are, respectively: (i) that the stimulant effects of pre-loading with cocaine make persistent responding subsequently impossible to interpret, (ii) that LiCl does not readily devalue (through illness) a non-ingested reinforcer and (iii) that contingency degradation (delivering ‘free’ reinforcers when an animal is otherwise responding for drug) is very difficult in the context of the several minutes of duration of the effect of a self-administered cocaine reinforcer (Everitt & Robbins, 2013).

However, rats responding for oral cocaine or alcohol more rapidly become resistant to devaluation by LiCl-induced malaise, i.e. habitual drug seeking behaviour is instantiated more readily than habitual responding for a food reinforcer (Dickinson *et al*., 2002; Miles *et al*., 2003). Moreover, in rats responding for alcohol, it was not only confirmed that development of resistance to reinforcer devaluation induced by specific satiety (free access to alcohol before the instrumental seeking test) occurred more rapidly than was the case for a food reward, but that this was accompanied by a shift from control by the DMS to the DLS; thus, DMS inactivation prevented the effect of reinforcer devaluation early in training, thereby revealing its control by an A-O process, but inactivation of the DLS after 8 weeks of training resulted in restored sensitivity to reinforcer devaluation, thereby demonstrating devolved control over habitual alcohol seeking behaviour to the DLS (Corbit *et al*., 2012).

In rats seeking cocaine, additional evidence supports the hypothesis that seeking behaviour is initially goal-directed, but after extended training becomes habitual and under the control of the aDLS. Having developed a cocaine seeking–taking chained schedule, we initially showed that after limited training, seeking behaviour was sensitive to reinforcer devaluation (extinguishing the taking link of the chain) and therefore goal-directed (under A-O control) (Olmstead *et al*., 2001). Adopting the same methodology, it was shown subsequently both that cocaine seeking behaviour was insensitive to devaluation after extended training and that it depended on the aDLS, as inactivation of the DLS at this time reinstated sensitivity to devaluation (Zapata *et al*., 2010).

This now substantial literature has revealed much about the neural and psychological basis of the seeking of addictive drugs over time and the influence of pavlovian drug-associated CSs. The data are readily aligned with a fundamental understanding of goal-directed and habitual instrumental behaviour and the shifts in the relative dominance of one or other parallel learning process between early and late performance, an understanding that is complemented by other experimental and clinical data. Thus, prolonged but not brief stimulant self-administration alters markers of cellular plasticity in dorsal striatal neurons (Jedynak *et al*., 2007), as well as adaptations in striatal D2 DA receptors and metabolic markers that are perhaps the clearest demonstration of the way in which cocaine's effects spread from ventral to dorsal striatum the longer the drug is self-administered (Letchworth *et al*., 1997, 2001; Porrino *et al*., 2004). The escalation of drug intake in rats with extended cocaine access has further been shown to be under the control of micro-RNAs in the dorsal striatum (Im *et al*., 2010; Jonkman & Kenny, 2013). Cocaine administration accelerates the emergence of habitual responding for food (Nelson & Killcross, 2006) and also the shift from ventral to dorsal striatum of neuronal firing evoked by presentation of a pavlovian CS (Takahashi *et al*., 2007). In a T-maze, food-reinforced task, a transition was demonstrated in electrophysiological activity from the DMS, which was present during acquisition and early performance, but decreased with over-training to be dominated by DLS activity mediating habitual performance (Thorn *et al*., 2010). In mice, chronic intermittent alcohol vapour exposure resulted in impaired pavlovian influences on instrumental behaviour, a loss of CB1-receptor-mediated long-term depression in the DLS and the facilitation of DLS-dependent learning that indicated an increased propensity for the greater control over behaviour by the DLS in the progression to alcoholism (DePoy *et al*., 2013).

In imaging studies, cue-induced craving in humans addicted to cocaine has been shown to be associated with increased DA release and metabolic activity in the dorsal striatum (Garavan *et al*., 2000; Volkow *et al*., 2006). In alcohol-dependent, as compared with recreational alcohol drinkers, presented with alcohol-related CSs a shift in activation from the ventral to the dorsal striatum has also been demonstrated (Vollstaedt-Klein *et al*., 2010). Perhaps most intriguing is the finding that the left putamen was siginificantly enlarged both in cocaine-dependent individuals and in their siblings, who were not abusing cocaine (Ersche *et al*., 2013a,b). In a related study (Bohbot *et al*., 2013) human subjects learned a virtual maze task that can dissociate spatial and S-R response navigational strategies; response learners had increased dorsal striatal grey matter volume and activity measured using functional magnetic resonance imaging, while spatial learners had increased hippocampal grey matter and activity. It was further shown that the response learners had greater use of abused substances than spatial learners, including double the lifetime alcohol consumption, a greater number of cigarettes smoked and a greater lifetime use of cannabis. These data, especially the sibling study of Ersche *et al*. (2012a,b), indicate that greater dorsal striatal (caudate or putamen) volume is associated with a predisposition for drug use and part of an endophenotype that may predict a greater propensity to acquire drug seeking habits.

The notion of the parallel and serial operation of corticostriatal loops in motivational, pavlovian and instrumental processing raises the issue of how, mechanistically, the loops interact. The reinforcing effects of many abused drugs, but especially stimulants, depend upon dopaminergic transmission in the NAcb shell and closely related ventral striatal areas such as the olfactory tubercle (Di Chiara & Imperato, 1988; Ikemoto & Wise, 2004). Pavlovian influences on instrumental behaviour depend upon the NAcb core and its afferents from the amygdala (Hall *et al*., 2001; Cardinal *et al*., 2002; Corbit & Balleine, 2005). Instrumental A-O behaviour depends upon the pDMS and its connections with the OFC, as well as on the prelimbic prefrontal cortex (PFC) (Killcross & Coutureau, 2003; Ostlund & Balleine, 2005; Yin *et al*., 2005b; Corbit *et al*., 2012; Murray *et al*., 2012a), while S-R habits depend upon the aDLS and its connections, presumably, with the sensorimotor and motor cortex, as well as on the infralimbic PFC (Killcross & Coutureau, 2003; Yin *et al*., 2004; Corbit *et al*., 2012; Murray *et al*., 2012a). The complex product of these interactions underlies the learning and performance of goal-directed drug seeking and its eventual subordination by automatic S-R processes, as well as the impact on both of drug-associated CSs. We have recently presented data and reviewed the evidence (Belin *et al*., 2013; Everitt & Robbins, 2013) suggesting that these interactions and transitions between ventral and dorsal striatal domains depend in a significant way upon the recurrent circuitry that, via projections to midbrain DA neurons, allows ventral striatal processing to influence DA-dependent processing in the dorsal striatum (Haber *et al*., 2000) – indeed, links the NAcb shell to the core, to the DMS and ultimately the DLS (putamen in primate brains) via connections with progressively more lateral midbrain DA neurons – from medial ventral tegmental area to lateral substantia nigra (Fig. 2). Disconnecting the NAcb core from DA transmission in the DLS (by making a unilateral, cell body-specific lesion of the core and infusing the DA receptor antagonist flupenthixol into the contralateral DLS, thereby disabling this system bilaterally) significantly reduced well-established, or habitual cocaine seeking at a time point when seeking dependent on the DLS, yet this procedure had no effect on a newly acquired (goal-directed) instrumental food seeking response (Belin & Everitt, 2008). In a closely related *in vivo* neurochemical study, it was further shown in rats self-administering cocaine that late-developing drug CS-evoked DA transients in the aDLS were completely prevented by an NAcb core lesion, providing direct evidence in a behavioural setting that DA release in the aDLS depended on antecedent processing in the NAcb core (Willuhn *et al*., 2012).

Together these data are consistent with drug-induced alteration of the striatal mechanisms predicted by the reinforcement learning model that assigns the role of ‘critic’ to the NAcb through outcome value learning for the purpose of prediction, which drives the ‘actor’, a role assigned to the DLS that mediates action selection (O'Doherty *et al*., 2004). According to this computational view, increased functional coupling of the NAcb and the aDLS, such as we have suggested may occur as the result of repeated drug taking, may reflect a failure in the critic properly to direct action selection in the actor, so rendering choices and actions rigid and independent of the value of outcomes (Dezfouli *et al*., 2009; Piray *et al*., 2010; Belin *et al*., 2013). Such increased functional coupling between the ventral and the dorsal striatum has recently been shown in former heroin addicts alongside a decrease in functional coupling between the striatum and the PFC (Xie *et al*., 2014).

There is a seeming paradox to be resolved in linking conditioned reinforcement, and indeed other pavlovian processes that depend critically on the amygdala, with the habit system represented by DLS circuitry, as the amygdala does not project directly to the DLS. How then do drug-associated CSs engage and support aDLS-dependent drug seeking habits? There are two main routes by which such interactions could be mediated: (i) BLA glutamatergic projections to the NAcb core (Wright *et al*., 1996) can engage the spiralling dopaminergic circuitry linking the core with the DLS (Fig. 2); (ii) the central amygdala (CeN) receives a major projection from the BLA and in turn projects directly to substantia nigra DA neurons that innervate the DLS (a system demonstrated to mediate pavlovian conditioned orienting; Han *et al*., 1997; Fig. 2). Using another variant of the disconnection approach, we have shown that disconnecting either the BLA or the CeN from DLS DA transmission (by making a unilateral lesion of either CeN or BLA in combination with either a contralateral infusion of flupenthixol into the aDLS) greatly decreased cue-controlled cocaine seeking habits (Murray *et al*., 2013b).

Thus, two converging systems may underlie the development and maintenance of DLS DA-dependent cocaine seeking habits, the first linking the BLA to the DLS via the NAcb core and spiralling loop circuitry, the second linking the CeN to the DLS via direct projections to nigral DA neurons. These circuitries may thereby enable the associative, motivational and general arousal properties of drug CSs to control habitual responding for cocaine. This dual control system has been further shown by our recent electrophysiological investigations that have demonstrated that while BLA neurons cannot directly drive the activity of DLS medium spiny neurons, they can incrementally and decrementally modulate the firing of medium spiny neurons driven by activation of their afferents from the motor cortex. Blockade of glutamate receptors in the NAcb core prevented the BLA gating of motor cortex-evoked DLS medium spiny neuron firing, providing direct evidence of the ability of BLA processing to influence activity of the DLS habit system through its glutamatergic interactions with the NAcb (Belin-Rauscent *et al*., 2013).

## The transition from drug seeking actions to habits – summary

There is no doubt that drugs are initially taken, then repeatedly taken, because of their reinforcing or rewarding, including subjective, effects. People want these drugs and will strive to attain them; rats and monkeys do the same. These drug seeking and taking actions depend upon the value of the drug and depend upon the pDMS system; they are also influenced by pavlovian drug-associated CSs mediated by the interacting amygdala–NAcb core system. With repetition and under real world conditions in which seeking behaviour is not always or reliably followed by the opportunity to take the drug, seeking habits are consolidated. Drug seeking habits are divorced from the value of the goal. They are elicited by drug CSs automatically, or implicitly, and do not depend on conscious awareness; they may often be performed in the face of higher cognitive processes, including the intention or decision not to take drugs. Habits are remarkably difficult to inhibit in the presence of the eliciting CSs. But it is also clear that drug CSs can induce subjective states of craving which are readily thought of as motivational states that can elicit drug seeking and taking as goal-directed responses. However, craving often correlates poorly with subsequent drug use (Ehrman *et al*., 1998; Tiffany, 2000) and can instead be understood as a *post-hoc* explicit rationalization of a mismatch between automatic cognitive schemata-induced drug seeking behaviour elicited by drug CSs in the absence of immediately available drug (Tiffany, 1990), as may be the case in some laboratory settings, including imaging studies.

We have suggested previously that drug seeking ‘incentive’ habits may explain apparent insight deficits associated with addiction, as they rely on the recruitment of a corticostriatal circuitry that aberrantly engages implicit rigid, or automatic, behavior by amygdala-dependent associative processes (Belin *et al*., 2013). However, the establishment of seeking and taking habits is not a sufficient explanation for the compulsive use of drugs that characterizes addiction; they more mark the beginnings of the loss of control over drug use as strongly conditioned drug-associated CSs occasion drug taking, as is seen with smoking after a meal, or in (more often outside, since smoking bans) a bar, or drinking after work with friends, often excessively, when the intention was not to do so. The devolution of control to a dorsal striatal habit system is a normal consequence of the frequent repetition of seeking and taking behaviour and is seen – indeed was first demonstrated – in animals responding for food reward (Adams & Dickinson, 1981). The development of habitual behaviour should not, then, itself be seen as aberrant, even though it seems to develop more rapidly for addictive drugs (alcohol and cocaine) than ingestive rewards. Automatizing behaviour is adaptive as it enables cognitive processes to facilitate rapid responses to altered contingencies in the environment, provided that in the face of those changing contingencies an individual can regain control over habitual behaviour. Thus, drug seeking habits and the underlying intrastriatal shifts in the control over behaviour alone should not be equated with addiction.

It is instead the loss of control over habitual drug seeking that may be the important event that leads to compulsive drug use, rather than the perhaps more widely held view of pathologically increased motivation for drugs, which remains rather difficult to reconcile with reductions in drug value through tolerance. Moreover, habit learning is potently influenced by stress (Dias-Ferreira *et al*., 2009), perhaps indicating that the stress associated with withdrawal may facilitate the establishment of drug seeking habits and that negative reinforcement, including negative conditioned reinforcement, may promote the ventral to dorsal shift in the striatal control over drug seeking. Understanding the associative structure underlying drug seeking and taking is not only of academic interest, but has therapeutic implications. For example, drug devaluation techniques may not be successful if drug seeking is habitual, as habits resist reinforcer devaluation. On the other hand, restoring reinforcer value may result in treatments such as cognitive behaviour therapy becoming more effective, as drug seeking and taking when goal-directed may then be more susceptible to motivational manipulations and the promotion of choice. It is therefore important to understand the basis of the loss of control over drug seeking habits and why it is that only some individuals lose this control whereas the majority do not.

We have suggested (Murray *et al*., 2013a) that a key aspect of this individual vulnerability results not from the effect of chronic exposure to drugs to facilitate intrastriatal shifts and the establishment of drug seeking habits, but in the nature of the habits themselves. Thus some vulnerable individuals may be characterized by more rigid corticostriatal circuitry such that once the control over drug seeking has devolved to the DLS, the now maladaptive drug seeking habit cannot be brought back under executive control, resulting in compulsive drug seeking.

## Compulsive drug seeking

However phrased, it is indeed compulsive drug use that is widely seen as a core aspect of addiction, and by that is meant repeated, persistent use, despite placing an individual in danger, compromising their health, family and social lives. The new iteration of the Diagnostic and Statistical Manual (DSM V; American Psychiatric Association, 2013) also recognizes that not everyone is vulnerable to developing substance-related disorders, and that it is low levels of self-control that may cause persistent use. Suboptimal self-control may be a predisposing factor for, or be the result of, excessive drug intake (Jentsch & Taylor, 1999) – the latter perhaps interacting with spontaneously occurring vulnerability traits (Belin *et al*., 2008).

There have long been drug-based explanations of compulsion. First and foremost is the view that drugs are repeatedly taken to avoid or postpone the aversive consequences of withdrawal through negative reinforcement (Wikler, 1973; Koob & Le Moal, 2001). Koob *et al*. (2004) have amassed a large body of data showing the importance of this mechanism in the escalation of drug intake in animals either given long access to cocaine or heroin, or made dependent on alcohol in vapour chambers (Ahmed *et al*., 2002; Koob *et al*., 2004; Gilpin & Koob, 2008). The withdrawal state is associated with raised reward thresholds, activation of stress systems in the amygdala and the development of hedonic allostasis – a pathological alteration in the set point of a hedonically positive, or at best neutral, state that an individual dependent on drugs persistently seeks but fails to achieve through repeated drug taking (Koob, 1996, 2008). As discussed above, while this view appeals to a negative affective state and aversive motivational processes, these may also profoundly influence the development of S-R drug seeking habits, although this has never been studied in an addictive drug context.

Koob & Le Moal (2005b) refer to this to this as the ‘dark side’ of addiction, but its light side counterpart involves the incremental responses to addictive drugs and drug-associated stimuli (sensitization; Robinson & Berridge, 1993) that result from intermittent, early drug exposure (and, in the majority of experimental studies, this exposure is non-contingent and administered by an experimenter, rather than being self-administered). It is best demonstrated for stimulants such as cocaine and amphetamine, but has also been shown with most addictive drugs and to be mediated by an upregulation of DA transmission in the NAcb (Robinson *et al*., 1988). The most common interpretation of the consequence of a sensitized DA system is a pathologically elevated, positive motivational state in which drugs are excessively wanted, excessively sought and repeatedly taken (Robinson & Berridge, 1993). These are subjective states that are indeed reported by humans who abuse or are addicted to drugs, but again they do not preclude the enhancement of implicit processes and the aberrant engagement of S-R habit learning. This has clearly been demonstrated in animals responding for food, in which even brief stimulant drug pre-exposure enhances dorsal striatal DA transmission and renders instrumental behaviour rapidly resistant to reinforcer devaluation (i.e. habitual) (Nelson & Killcross, 2006). Escalated cocaine intake also enhances the propensity of rats to display stereotypies that depend on dorsal striatal DA, and to show changes in dendritic spines, a marker of plasticity, in the dorsal striatum (Ferrario *et al*., 2005). Thus, the consequences of stimulant exposure are not confined to a NAcb-based incentive system, but instead progressively spread to the more dorsal and cognitive territories of the striatum.

There are influential and informative reviews of allostatic and sensitization theories and these will not be discussed further here (Robinson & Berridge, 2008; Koob & Volkow, 2010) (and see above). Instead, focus will be placed on the evidence suggesting that it is another, major consequence of addictive drug exposure that is causal in the development of compulsive drug use, namely prefrontal cortical dysfunction and the consequential loss or diminution of executive, or inhibitory, control over drug use (Jentsch & Taylor, 1999; Everitt & Robbins, 2005; Kalivas & Volkow, 2005; Volkow & Li, 2005; Baler & Volkow, 2006; Schoenbaum & Shaham, 2008; Torregrossa *et al*., 2008; Ersche *et al*., 2013a). Experimentally, even relatively brief exposure to stimulants, including their self-administration, can result in impairments in tasks that depend on the OFC, for example perseverative deficits in reversal learning (Calu *et al*., 2007) that were correlated with altered responses of OFC neurons to cues predicting outcomes (Takahashi *et al*., 2007). Perseveration in reversal learning is a form of compulsive behaviour that has also been seen in chronic stimulant abusers and shown to correlate with reduced activity in the anterior caudate nucleus (Ersche *et al*., 2008, 2012a). The impairment was ameliorated by treatment with a D2 DA receptor agonist (Ersche *et al*., 2011b), a finding of particular importance since cocaine (as well as alcohol, methamphetamine and heroin) abusers have reduced levels of D2 DA receptors in the striatum, which are correlated with OFC functional hypoactivity (Volkow *et al*., 1993, 2001), indicating that the enhancement of D2 DA signalling may have restored function in the OFC–caudate system. In addition to probabilistic reversal learning deficits, cocaine abusers have been reported to show impairments in a variety of behavioural and cognitive functions, including poor behavioural adjustment to environmental contingencies (Bechara, 2005) and decision-making deficits in a gambling task (Rogers *et al*., 1999; Ersche *et al*., 2005) that are indicative of OFC dysfunction, as they are also seen following OFC damage (Rogers *et al*., 1999).

Chronic drug abuse may therefore be causal in inducing suboptimal prefrontal cortical, including OFC and anterior cingulate cortex, function (Volkow & Fowler, 2000; Kaufman *et al*., 2003; Hester & Garavan, 2004) that is reflected in reduced inhibitory control and deficits in decision-making. That PFC dysfunction is more a consequence of chronic drug use than a vulnerability trait leading to drug abuse through making poor decisions, or a lack of sensitivity to the consequences of decisions to engage in drug taking (Ersche *et al*., 2012a,b) is strongly indicated by an endophenotype study of non-drug-abusing stimulant abusers (Ersche *et al*., 2013a). Changes in OFC and anterior cingulate cortex grey matter seen in stimulant abusers were not observed in the siblings, where instead there were increases in the medial temporal lobe grey matter and putamen (as discussed above) and reductions in the superior temporal gyrus, post-central gyrus and insula. Moreover, a group of recreational drug users in the same study showed increased OFC grey matter volumes, which may therefore be related to an enhanced ability to maintain control over infrequent drug use (Ersche *et al*., 2013a). These data strongly indicate that prefrontal cortical grey matter changes reflect, rather than pre-date, drug abuse and support the hypothesis that compulsive drug use is at least in part caused by chronic drug effects leading to impaired top-down control by prefrontal cortical processes (Jentsch & Taylor, 1999; Goldstein *et al*., 2004; Everitt & Robbins, 2005).

Further supportive data include the observation that stimulant-dependent subjects had elevated scores on the OCDUS (Obsessive Compulsive Drug Use Scale) scale, which both measures compulsive drug use and captures behavioural predispositions in drug taking, rather than reflecting subjective motivational responses based on ‘liking’ or ‘wanting’ (Ersche *et al*., 2010, 2011a,b). Furthermore, stimulant abusers and their siblings tend to show obsessive-compulsive-like or ritualistic behaviours which Ersche *et al*. (2012b) have suggested may indicate an overlap between obsessive-compulsive disorder (OCD) and compulsive drug use in addiction, reflecting some commonality in underlying prefrontal cortical–striatal mechanisms (Meunier *et al*., 2012; Robbins *et al*., 2012).

Experimental animal models of addiction have always faced a major challenge when trying to capture compulsive drug use as they require chronic, not acute, drug self-administration (and therefore, in the case of cocaine or heroin, long-term maintenance of i.v. catheters) and also should reveal individual differences in the propensity to lose control over drug seeking and taking. Not every individual who takes drugs loses that control and compulsively uses drugs despite the adverse or aversive consequences of doing so – a proportion that across drugs and studies has been estimated to be around 20–30% (Anthony *et al*., 1994). However, behavioural paradigms have been established, especially in animals self-administering cocaine, that do measure compulsive drug seeking and taking. Initially, we showed using a seeking–taking chained schedule that a chronic, but not a brief, history of cocaine self-administration resulted in the greatly reduced ability of a fear-conditioned CS (established by Pavlovian fear conditioning) to suppress cocaine seeking (Vanderschuren & Everitt, 2004). This loss of fear-conditioned suppression of cocaine seeking was not associated with any change in fear behaviour or conditioning and so indicated that after chronic drug use, rats were prepared to risk seeking cocaine in the presence of a CS that signalled an aversive outcome. However, all rats having chronically self-administered cocaine showed this change, just as all rats exposed relatively briefly to stimulant showed impaired reversal learning and an increased tendency to acquire S-R habits. Thus, while this experiment demonstrated an effect of chronic cocaine on later sensitivity to danger signals – albeit specifically in a cocaine seeking context and not generally – it did not reveal individual propensities to show this effect in a vulnerable sub-population.

**FIG. 3:**
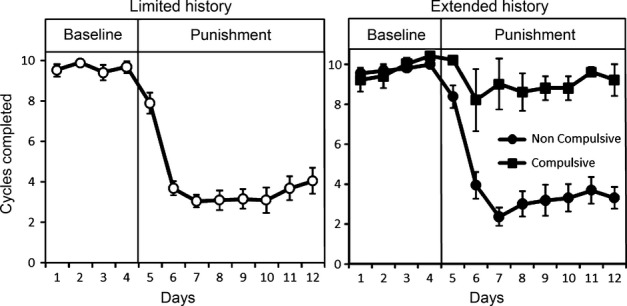
Compulsive cocaine seeking in 20% of vulnerable rats after an extended cocaine taking history. Rats were trained in a cocaine seeking-taking chained schedule of reinforcement, completing around 10 ‘cycles’ of seeking and taking per session (a cycle is comprised of a variable 2 minute interval of seeking responses, followed by the opportunity to self-administer cocaine by responding on the taking lever; see Pelloux *et al*., 2007). Shown in both left and right panels are seeking cycles on 4 baseline days, at which point intermittent and unpredictable punishment was introduced to terminate 50% of seeking cycles; on the remaining 50% of cycles, pressing the taking lever resulted in an i.v. infusion of cocaine. Left Panel: After a limited history of cocaine self-administration, all rats suppressed their cocaine seeking when the intermittent punishment contingency was introduced. Right Panel: A sub-group of rats, 20% of the population, persisted in seeking cocaine in the face of punishment, i.e. were compulsive, whereas the majority suppressed their cocaine seeking (Pelloux *et al*., 2007). This result has been replicated in different laboratories, using the same or slightly different procedures and in different strains of rats (Deroche-Gamonet *et al*., 2007 Pelloux *et al*., 2007; Belin *et al*., 2007; Cannella *et al*., 2007; Chen *et al*., 2007). Data from Pelloux *et al*., 2007.

Two procedures have, however, captured the vulnerability to develop compulsive drug use, defined as seeking cocaine in the face of aversive or negative outcomes. In the three addiction-like criteria model (Deroche-Gamonet *et al*., 2004; Belin *et al*., 2008), 20% of rats having self-administered cocaine for 100 days (but not 40 days) continued to respond for cocaine even though receiving punishment (mild footshock) for doing so; they also persisted in responding when an environmental stimulus signalled that cocaine was not available and an increased motivation for cocaine (assessed under a progressive ratio schedule). This procedure therefore captured the notion of vulnerability to develop compulsive cocaine taking behaviour. In our approach (Pelloux *et al*., 2007, 2012; Jonkman *et al*., 2012a), the seeking–taking chained schedule described above was adapted so that on 50% of trials the outcome of seeking responses was the opportunity to take (i.e. self-administer) cocaine, but unpredictably on 50% of trials in a session, the outcome of seeking was mild footshock punishment and not access to the taking lever. Therefore, rats had to run the risk of punishment when seeking the opportunity to take cocaine. After a brief cocaine history, all rats ceased seeking cocaine when the punishment contingency was introduced and could therefore be considered as voluntarily abstaining from drug seeking. However, after a long history of cocaine self-administration, whether or not they escalated their cocaine intake, around 20% of rats continued to seek cocaine, while 80% abstained (Fig. 3). This compulsive cocaine seeking was seen in rats regardless of any increased motivation for the drug, suggesting that some forms of compulsion, as operationalized in preclinical models, may develop without coincident increases in motivation. The extent of exposure to cocaine, rather than the degree of conditioning through pavlovian pairings of CS and drug, was further shown to be a critical factor in determining the development of cocaine seeking under punishment (Jonkman *et al*., 2012b). Moreover, the propensity to show compulsive cocaine seeking in a therefore vulnerable sub-population has now been demonstrated in different strains of rats and in different laboratories (Deroche-Gamonet *et al*., 2004; Pelloux *et al*., 2007; Belin *et al*., 2008; Cannella *et al*., 2013; Chen *et al*., 2013) (but see Waters *et al*., 2014) using either the three-criteria or seeking–taking chained schedule procedures. These behavioural approaches to studying addiction in animals have enabled investigation of the neural basis of compulsive drug seeking and also the identification of individuals who are vulnerable to compulsivity when having self-administered cocaine over long periods. It is further becoming clear that these behavioural approaches can be adapted to study the compulsive seeking of heroin, alcohol and high incentive food.

In having hypothesized that there is a shift in the balance of control over drug seeking from PFC to striatum as drug seeking becomes compulsive – conceptualized as the ‘must do’ (must seek and take drugs) of a compulsive habit, rather than the ‘must have’ of an increased goal-directed motivational state (Everitt & Robbins, 2005) – recent data have shown the involvement of a discrete aDLS domain and altered PFC function in compulsive cocaine seeking in rats. Inactivation of the aDLS zone selectively disrupted punished, but did not affect unpunished, drug seeking even after extended training, whereas inactivation of an adjacent mid-lateral striatal area disrupted drug seeking regardless of the stage of training, whether punished or not. Since the effect of inactivating the aDLS under punishment conditions was present before delivery of the first punishment in a session, we argued that taking the aDLS offline enhanced the influence of recalled negative consequences of cocaine seeking (Jonkman *et al*., 2012a). These results indicated that the aDLS selectively mediates the rigidity of responding after over-training and under the threat of punishment, while the midlateral striatum with its motor cortical connectivity is instead necessary for instrumental responding *per se*, whether punished or not.

Pelloux *et al*. (2012) showed that the sub-population of rats that continued to seek cocaine despite punishment had markedly reduced levels of 5-HT utilization across prefrontal cortical areas (as well as decreased DA utilization in the dorsal striatum), whereas the 80% of rats that became abstinent after the introduction of the intermittent shock contingency did not. Since both groups had very similar total levels of cocaine exposure, this result revealed an interaction between some individual characteristic(s) of the 20% vulnerable rats and a long-term cocaine taking history (Pelloux *et al*., 2012). That the low levels of 5-HT utilization were causal in compulsive cocaine seeking was demonstrated by showing that forebrain 5-HT depletion, or systemic treatment with a 5-HT2C receptor antagonist, after a short cocaine history, when none of the rats was compulsive, resulted in increased levels of seeking under punishment. Moreover, treatment with a serotonin-selective 5-HT reuptake inhibitor (SSRI), citalopram, dose-dependently reduced compulsive seeking in rats that had developed this behaviour after a long drug taking history (Pelloux *et al*., 2012). The latter finding indicates the potential use of a relatively high dose of an SSRI to reduce compulsive cocaine seeking in patient groups that are characterized by the compulsive nature of their cocaine use (Ersche *et al*., 2011a).

In a recent noteworthy study that adopted our cocaine seeking–taking task with intermittent punishment, our earlier demonstration of a compulsive cocaine seeking sub-population after long access was replicated (Chen *et al*., 2013). In addition, *in vivo* optogenetic stimulation of the prelimbic cortex in compulsive animals resulted in decreased compulsive cocaine seeking and increased latencies to seek. By contrast, the 80% sub-population of rats that had suppressed their cocaine seeking during punishment subsequently increased their cocaine seeking under punishment, i.e. became compulsive, after optogenetic inhibition of the prelimbic cortex (Chen *et al*., 2013). In the context of the demonstration that pre-training, pre-cocaine experience, lesions of the same prelimbic cortical area did not result in compulsive cocaine seeking in rats after a limited cocaine history (Pelloux *et al*., 2013), these data further suggest that impaired prefrontal (prelimbic in this case) cortical function is not a vulnerability characteristic leading to compulsive cocaine use, but an emergent characteristic that is a consequence of long-term cocaine use. It will be of great interest in the future to link these prefrontal cortical, dorsal striatal and neurochemical mechanisms that have been demonstrated individually to be causally related to compulsive drug seeking in rats after a protracted cocaine taking history.

## Impulsivity and the vulnerability compulsively to seek cocaine

Impulsivity has long been recognized as a characteristic of individuals addicted to drugs, along with sensation seeking and poor decision-making (Koob & Le Moal, 2005a; Kreek *et al*., 2005; Dom *et al*., 2006; Verdejo-Garcia *et al*., 2006; Zilberman *et al*., 2007; Ersche *et al*., 2010). It has now been established that impulsivity may be causally linked to the loss of control over drug use, most especially stimulant use, rather than being a consequence of taking drugs such as cocaine and amphetamine (Dalley *et al*., 2007; Belin *et al*., 2008; Ersche *et al*., 2011a). Rats that were impulsive on a five-choice serial reaction-time task (5CSRTT), which measures a form of motor or ‘waiting impulsivity’ (HI rats), escalated their cocaine intake in long access sessions to a degree that was much greater than low impulsive (LI) rats or the general population of rats, although they did not acquire cocaine self-administration more readily (Dalley *et al*., 2007). This is one of several important points of contrast to so-called ‘high responder’ (HR) rats (i.e. rats that show a greater locomotor response to a novel inescapable environment, which is not the same as sensation seeking) that acquire cocaine or amphetamine self-administration at very low doses of drug which are not self-administered by LR rats, or the general population of rats (Piazza *et al*., 1989; Dalley *et al*., 2007). Moreover, HR rats, showing an increased propensity to acquire cocaine self-administration are greatly disrupted in this behaviour by a concurrently available ingestive reinforcer (N. Vanhille and D. Belin, unpublished data), perhaps indicating a model of the propensity to use drugs recreationally. The HI rats that lose control over cocaine intake constitute 8–14% of the Lister-hooded rat population used in our laboratory. When given an extended period of access to cocaine, HI but not HR rats developed compulsive cocaine seeking that persisted despite punishment (Belin *et al*., 2008) and showed a greater propensity to relapse after abstinence (Economidou *et al*., 2009). The latter finding is of particular interest as it had earlier been shown that the self-administration of cocaine reduced impulsivity in HI rats, but that HI re-emerged at some point during 3 weeks of withdrawal (Dalley *et al*., 2007), thereby suggesting that the effective treatment of impulsivity in individuals trying to maintain abstinence after cocaine use might enhance their chances of success in doing so (de Wit & Richards, 2004).

The link between pre-existing impulsivity and the subsequent loss of control over drug intake and compulsivity that develops in rats given access to cocaine is not seen in rats given access to heroin (McNamara *et al*., 2010), or in alcohol-preferring P rats (Y. Pena-Oliver, C. Giuliano, J.D. Dalley and B.J. Everitt, unpublished data), but there is a predictive relationship between impulsivity and the escalation of nicotine self-administration (Diergaarde *et al*., 2008), the tendency to over-eat palatable food (Diergaarde *et al*., 2009; Velázquez-Sánchez *et al*., 2014) and to develop another form of compulsivity, schedule-induced polydipsia, or SIP (Ansquer *et al*., 2013). Thus, impulsivity may be a causal trait that predisposes individuals to develop compulsive behaviours, including the compulsive use of stimulants and also high-incentive foods. Evidence for this hypothesis in humans comes from endophenotype studies (Ersche *et al*., 2010, 2013a) showing that impulsivity was significantly increased in a stimulant abusing group and also, although to a lesser extent, in their siblings compared with controls. Sensation seeking was not significantly greater in the siblings, but was increased in the stimulant abusing group (Ersche *et al*., 2013a). These data indicate that impulsivity in substance abusers may therefore reflect a combination of pre-existing and drug-induced influences whereas sensation-seeking, but not novelty seeking [see Arnett factorial analysis of SS scale (Arnett, 1994; Belin *et al*., 2011)] may be more related to drug-induced rather than genetic effects. The accumulated data are of major importance with regards to the notion of vulnerability to addiction because they highlight the dissociable factors that contribute to the motivation underlying initiation of drug use from those related to the transition to compulsive drug use. While both are present in those who become addicted to drugs, it is the latter that should be the increasing focus in studies trying to understand the mechanisms of addiction.

The neural mechanisms underlying impulsivity are increasingly understood (reviewed by Dalley *et al*., 2011), and so will not be considered in detail here. In summary, a growing body of data has implicated the NAcb in impulsivity in the 5CSRTT, especially its DA afferents from the ventral tegmental area and glutamatergic afferents from the medial PFC and BLA. The NAcb is also implicated in impulsivity measured in delay discounting tasks and rats that are impulsive in the 5CSRTT show an increased preference for small immediate over larger delayed rewards. HI rats have significantly reduced availability of DA D2/3 receptors in the ventral striatum, including the NAcb, but not in the dorsal striatum that correlated with their level of impulsivity (Dalley *et al*., 2007). HI rats have further been shown to have reduced grey matter density and reduced glutamic acid decarboxylase in the left NAcb core, which together with evidence of reduced dendritic spine and microtubule markers, suggests an interplay between DA transmission and dendritic spines on medium spiny NAcb neurons in the expression of the motor impulsivity (Caprioli *et al*., 2014b) that is associated with escalation of cocaine intake and compulsive cocaine seeking.

We have recently investigated the relationship between high impulsivity and the establishment of habitual cocaine seeking as indexed by the transition to control over behaviour by DA in the aDLS, investigating whether HI predisposed rats to escalate cocaine intake and to develop compulsive cocaine seeking by facilitating S-R habit learning, or whether the emergence of S-R habits and compulsion are independent and co-occurring processes. This question is timely, especially in the context of misunderstandings about the psychological constructs of S-R habits and compulsion and the relationships between them. For example, although escalation of drug self-administration under low FR schedules – i.e. simple drug *taking* – is an index of the loss of control over drug intake, there are to date no data to suggest that this is associated with the establishment of a drug *seeking* habit. Yet this interpretation has been placed on the loss of phasic DA signaling in the ventral, but not dorsal striatum associated with the escalation of cocaine intake following long access (Caprioli *et al*., 2014a; Willuhn *et al*., 2014). In fact, a long history of drug taking under FR1 did not result in a shift to control over responding by the aDLS, yet the same degree of total cocaine exposure over the same time period did result in the shift when animals were seeking cocaine under a second-order schedule (Murray *et al*., 2012a). Thus, it is the interaction of drug seeking under the control of drug-associated CSs mediating delays and cocaine reinforcement that results in the establishment of drug seeking habits. Moreover, escalation of cocaine intake during extended access sessions has not systematically been shown to facilitate the emergence of compulsive cocaine seeking (Ahmed *et al*., 2003; Deroche-Gamonet *et al*., 2004; Pelloux *et al*., 2007; E. Ducret, M. Puaud, J. Lacoste, E. Dugast, A. Belin-Rauscent, J. Murray, B.J. Everitt, J.-L. Houeto and D. Belin, unpublished observations).

The results of our recent study showed that high impulsivity predicting both the escalation of cocaine SA and compulsive cocaine intake, was in fact associated with a delay, not a facilitation in the progressive shift in the control over cocaine seeking by aDLS DA-dependent processes (Murray *et al*., 2013). The delayed recruitment of the aDLS may be related to the reduced availability of DA D2/3 receptors in the NAcb of HI rats through an influence on cocaine-induced adaptations (Moore *et al*., 1998; Nader *et al*., 2002; Besson *et al*., 2013) shown to progress from ventral to dorsal striatum over extended periods of cocaine self-administration. Furthermore, the progressive decrease in DA D2 receptors and messenger mRNA levels seen in primates (Nader *et al*., 2002) and rats (Moore *et al*., 1998; Besson *et al*., 2013) following extended cocaine self-administration was also delayed in HI compared with LI rats (Besson *et al*., 2013), despite lower baseline levels of DA D2 mRNA in the NAcb of HI rats (Dalley *et al*., 2007; Besson *et al*., 2013). The delayed recruitment of the aDLS to control habitual cocaine seeking in HI rats may therefore be attributed to the remediation of low DA D2 receptors in the NAcb by self-administered cocaine that has the additional effect of decreasing impulsivity (Dalley *et al*., 2007; Caprioli *et al*., 2013).

In psychological terms, the low availability of DA D2 receptors in the NAcb of vulnerable HI rats may result in prolonged dominance of control over cocaine self-administration by ventral striatal circuitry, leading to the escalation of cocaine intake. Hence the contributions of impulsivity and habits to addiction are dissociable. The propensity to develop S-R habits, which in itself is not an aberrant process but the natural consequence of the repetition of instrumental behaviour under conditions of delayed and unpredictable rewards, is distinct from the inability to regain control over maladaptive habits that have become inflexible, such as those that are seen in addicted individuals who compulsively seek and take drugs. It is the rigid nature of drug seeking habits and the associated difficulty in regaining inhibitory control over them that is a core feature of addiction (Belin *et al*., 2013). The rigidity of maladaptive drug seeking habits can then be seen as stemming from a neurobiological rigidity, either in prefrontal or in striatal areas, perhaps indexed by impaired plasticity in the NAcb (Kasanetz *et al*., 2010).

## Towards new treatments for addiction

Despite the increased understanding of the neural mechanisms of drug reward, drug seeking and taking, compulsivity and vulnerability to addiction and relapse, there has been rather limited progress in utilizing the wealth of preclinical and clinical data to develop new treatments. The introduction of harm reduction approaches has been of major importance, including methadone and nicotine replacement treatment for heroin and nicotine addiction, respectively, and the more recent approval of the opioid receptor antagonist nalmephene to reduce the volumes of alcohol drunk by those abusing alcohol (Soyka, 2014). However, the data reviewed above and more suggest that it should be possible to develop treatments that promote abstinence and decrease the probability of relapse in those trying to maintain an abstinent state. There are different targets in this regard, for example to reduce the impact of stress and anxiety associated with relapse to alcohol use, or to medicate dysphoric and anhedonic states that can persist long into withdrawal from stimulants, nicotine and opiates (Koob & Le Moal, 2005a). The approach I wish to highlight here, however, is prevention of relapse by using treatments that diminish the impact of drug-associated stimuli on drug seeking – although this is not to underestimate the impact of drug contexts, reviewed elsewhere (Crombag *et al*., 2008; Badiani, 2013; Bossert *et al*., 2013).

There are abundant data, some reviewed above, showing that drug CSs can evoke craving and relapse, as well as activation of limbic cortical structures including the amygdala, OFC and ventral striatum. Drug CSs can also elicit S-R habits and relapse through implicit processes associated with the dorsal striatum. The impact of drug CSs on limbic cortical–striatal circuitry, craving and relapse may be increased by the loss of prefrontal cortical gating processes which control the extent to which amygdala and hippocampal afferents interact with striatal neurons – interactions that are modulated by DA transmission (Goto & Grace, 2005). Reducing the impact of drug CSs on drug seeking is not a new experimental approach and extinction–reinstatement procedures have perhaps been the most widely used in animal models of relapse (Stewart, 2004). Extinction in these models relates not to drug CSs, as used in cue exposure therapy (Conklin & Tiffany, 2002; Park *et al*., 2014), but to the instrumental behaviour of lever pressing, i.e. the drug taking response – extinction training that not only omits the drug, but also the drug-associated CS. The reinstatement, or relapse, test is conducted by again presenting the drug CS, but not the drug, following performance of the recently extinguished lever press response (Shalev *et al*., 2002; Shaham & Miczek, 2003; Epstein *et al*., 2006; McNally, 2014).

This approach has generated much data on the neural circuitry of relapse, especially that involving interactions between the medial PFC, BLA and NAcb core and the marked alterations in glutamate transmission and homeostasis that underlie the operation of this neural circuitry and has been reviewed in detail elsewhere (Kalivas *et al*., 2006; Reissner & Kalivas, 2010; Bossert *et al*., 2013). While it is the case that this procedure provides a low baseline of responding from which to measure the CS- (or drug-, or stress-) induced increase in responding at the relapse test, it should be borne in mind that there is little in common with the establishment of abstinence and relapse in individuals addicted to drugs, including those in treatment programmes. There is rarely evidence of the extinction of drug taking behaviour (injecting placebo instead of drug, for example, although perhaps drinking non-alcoholic drinks might meet this criterion) and, since relapse is instantaneous following extinction, the indications are that extinguishing drug taking in the clinic will not be a very productive approach to treatment. However, these approaches have shown that presentation of the drug-associated CS response-contingently – i.e. as a conditioned reinforcer – rapidly reinstates lever pressing and a variety of pharmacological treatments have been seen to reduce the propensity for reinstatement (Bossert *et al*., 2005; Yahyavi-Firouz-Abadi & See, 2009; Bossert *et al*., 2013; McClure *et al*., 2014). However, it is difficult to disentangle effects of these treatments on extinction learning and retrieval from those that specifically reduce the behavioural effect of drug CS presentation. The interactions between extinction learning and conditioned reinforcement are likely to be complex and are poorly understood.

Increasingly, simply imposing a drug-free period (without instrumental extinction) is used in relapse procedures, so-called enforced abstinence, and these have revealed the phenomenon of ‘incubation’ – often called ‘incubation of drug craving’ – whereby the impact of the drug CS acting as a conditioned reinforcer actually grows or strengthens with increasing durations of the abstinence period, a phenomenon dependent upon a key neuroadaptation in the amygdala (Grimm *et al*., 2001; Lu *et al*., 2004; Pickens *et al*., 2011; Marchant *et al*., 2013).

In contrast, the approach we have taken is to exploit the first drug-free period of drug seeking under a second-order schedule of reinforcement, a period when behaviour depends critically on response-contingent presentation of drug CSs (Arroyo *et al*., 1998; Everitt & Robbins, 2000) as well as anticipation of the first drug infusion of the day (Pilla *et al*., 1999; Di Ciano & Everitt, 2003a; Di Ciano *et al*., 2003). The reasoning here is that if a pharmacological pre-treatment can reduce or prevent drug seeking, just as omission of drug CS presentations does, then this would indicate a treatment that can diminish drug CS-elicited and maintained seeking of drugs and thereby prevent relapse. Punishment of drug seeking resulting in voluntary abstinence has also been used, although infrequently, in studies of subsequent relapse (Economidou *et al*., 2009; Marchant *et al*., 2013).

There have been many findings indicating novel treatments for relapse prevention, often with effects across animal models of drug seeking and relapse. A DA D3 receptor (D3R) antagonist and a D3R partial agonist were perhaps the earliest and most promising, as they were seen to reduce cocaine seeking (Pilla *et al*., 1999; Le Foll *et al*., 2002; Di Ciano *et al*., 2003) and to prevent relapse in reinstatement and other procedures in animals responding for cocaine, nicotine and heroin (Heidbreder, 2013). An important characteristic of D3R antagonism is that it is not associated with debilitating motor or other extra-pyramidal side effects that probably prevent the use of DA D1 or D2 receptor antagonists clinically to prevent relapse, even though they prevent reinstatement in animal models. In clinical trials, a D3R antagonist has shown efficacy in treating methamphetamine addiction (Paterson *et al*., 2014). However, a new medication has not been approved for clinical use in the treatment of addiction.

The novel μ-opioid receptor antagonist GSK1521498 markedly reduced cocaine and heroin seeking (and in unpublished studies, also alcohol seeking – C. Giuliano, Y. Pena-Oliver and B.J. Everitt) under second-order schedules of reinforcement (Giuliano *et al*., 2012, 2013), effects that were significantly greater than those of naltrexone, which has been in clinical use for some years to treat alcohol dependence, and also to diminish cue-induced alcohol and cocaine craving (Volpicelli *et al*., 1992; Davidson *et al*., 1999; Palfai *et al*., 1999; Franck *et al*., 2008; Jayaram-Lindstrom *et al*., 2008). Here, then, is the potential to target a mechanism that is common to cue-induced drug seeking and relapse across addictive drug classes and with a novel compound that shows greater efficacy than one that is clinically available. However, a commonly held view is that opioid receptor antagonists have only a limited clinical utility – for example to prevent drinking in an alcoholic population—partly because of small effects and poor compliance. An important point to make here is that it seems very unlikely that drugs such as naltrexone, or the novel GSK1521498, will actually stop alcohol-dependent individuals from drinking (even though reducing amounts drunk), or those addicted to cocaine and heroin from using drugs; indeed, the experimental data show limited effects of opioid antagonism when drugs have been self-administered (Giuliano *et al*., 2013). But they might prevent relapse in individuals who have become abstinent by whatever means. What is needed are clinical trials that are undertaken in a more appropriate way in individuals who have, perhaps recently, become abstinent and to use treatments such as a D3R antagonist, a μ-opioid receptor antagonist – and other promising leads, including a hypocretin-1 receptor antagonist (Martin-Fardon & Weiss, 2014) or mGluR 2/3 receptor agonists (Hao *et al*., 2010) – to increase the likelihood of maintaining abstinence and preventing relapse occasioned by drug-associated stimuli. Of particular interest is the finding that the γ-aminobutyric acid (GABA)-B receptor agonist baclofen shown to reduce cocaine seeking in rats (Di Ciano & Everitt, 2003b) also reduced craving and associated limbic activations in human cocaine-dependent individuals and, most intriguingly, to do so in response to subliminal cocaine-associated cues (Young *et al*., 2014), suggesting that enhancing GABA transmission in this way might act also on implicit processes, such as those eliciting S-R habits.

A highly promising treatment has arisen following the work of Kalivas *et al*. (2006) targeting cocaine-induced alterations in glutamate homeostasis with *N*-acetyl cysteine (NAC), a cysteine pro-drug that is a substrate for the cysteine/glutamate antiporter. Having first been shown to prevent relapse in both cue and drug extinction–reinstatement procedures (Zhou & Kalivas, 2008; McClure *et al*., 2014), NAC has subsequently been shown significantly to reduce habitual (aDLS-dependent) cocaine seeking under a second-order schedule of reinforcement (Murray *et al*., 2012b) and to reduce compulsive cocaine seeking in rats displaying addiction-like behavior after an extensive cocaine taking history, enabling them better to withhold seeking responses even after the cessation of punishment, an effect associated with NAC-induced cellular plasticity in the aDLS (E. Ducret, M. Puaud, J. Lacoste, E. Dugast, A. Belin-Rauscent, J. Murray, B.J. Everitt, J.-L. Houeto and D. Belin, unpublished observations). Few other potential medications have been shown to have efficacy after both short- and long-term drug taking when the mechanisms controlling drug seeking have undergone the transition from action to habit to compulsion. The gradually accumulating clinical data show that NAC is well tolerated in humans and that it can successfully promote abstinence from cocaine, nicotine and cannabis use (McClure *et al*., 2014).

Despite these and many other therapeutic leads too numerous to recount here, it is apparent that at present we are in an era when many pharmaceutical companies do not have a focus on developing new neuropsychiatric medications in general, let alone medications specifically for addiction, these rarely having been given a high priority. It can only be hoped that this era will soon be over. There may, however, be another approach to addiction treatment which is to use drugs that are currently licensed and clinically approved to treat other neuropsychiatric disorders but instead to promote abstinence and prevent relapse in the treatment of addiction. For example, the noradrenaline selective reuptake inhibitor atomoxetine, which is used clinically in the treatment of attention deficit hyperactivity disorder (ADHD), markedly and dose-dependently reduced cocaine and heroin seeking and also prevented relapse in HI rats that showed a greater propensity to relapse after abstinence (Economidou *et al*., 2009, 2011). The latter finding may further indicate that treating impulsivity in abstinent stimulant abusers to prevent relapse will be particularly valuable (Dalley *et al*., 2007; Economidou *et al*., 2009; Caprioli *et al*., 2014b). Atomoxetine has also been shown to enhance the long-term extinction of cocaine seeking behaviour (Janak *et al*., 2012). Trials of atomoxetine in relapse prevention are clearly warranted and without the obstacles associated with introduction of a new medication.

Similarly, the SSRI citalopram dose-dependently reduced well-established cocaine seeking under a second-order schedule of reinforcement and reduced compulsive cocaine seeking (Pelloux *et al*., 2012). In experiments completed just before her untimely death, Daina Economidou showed that the effects of citalopram to reduce cue-controlled cocaine seeking were mediated by actions in the BLA and OFC, and interactions between these structures (Everitt & Economidou, 2011). It is, however, generally held that antidepressant medications, including SSRIs, have been shown to have limited effectiveness in the treatment of addiction where they have been used to medicate the depressed-like states of dysphoria and anhedonia seen in withdrawal (Covi *et al*., 1995; Grabowski *et al*., 1995; Batki *et al*., 1996). But that is not what is being proposed here. Instead, the relatively high doses of SSRIs used to treat OCD (Moeller *et al*., 2007), targeting the compulsive habitual nature of cocaine seeking are what might profitably be explored clinically (Vayalapalli *et al*., 2011; Pelloux *et al*., 2012), given the commonalities between addiction and OCD in terms of the underlying substrates of compulsion (Robbins *et al*., 2012; Everitt & Robbins, 2013).

These are but two examples of many in which novel uses of established and well tolerated neuropsychiatric medications could be investigated in well-conducted clinical trials of abstinence promotion in addicted populations. However, there is no doubt that issues of compliance (often stated to be a problem with μ-opioid and SSRI medications) must be addressed, as daily dosing would be required. Rarely have long-term treatment studies been conducted in animals and, in fact, these are extremely difficult given the limitations of the animal models of relapse currently employed.

## Modifying drug memories as a relapse prevention treatment

**FIG. 4:**
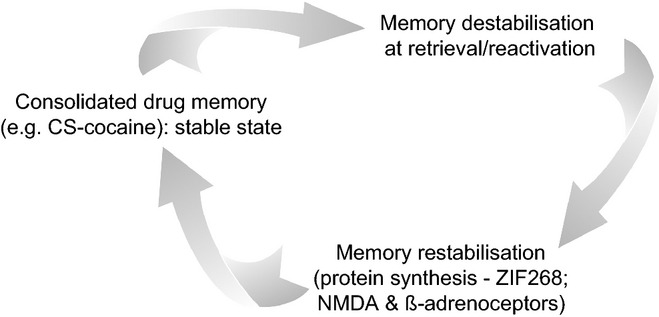
Illustration of the concept of memory reconsolidation. The consolidated drug memory, established by repeated Pavlovian association with an environmental stimulus (CS) and self-administered drug effect, is stored in a stable state. Brief presentations of the drug CS (called ‘reactivation’) can result in destabilization of the memory in the brain (in the case of a CS–drug memory, in the basolateral amygdala). The memory can persist in the brain if it is restabilized through *de novo* protein synthesis. The protein ZIF268 is a requirement of cued drug memory reconsolidation in the basolateral amygdala and is regulated by activation of NMDA receptors. Memory reconsolidation can be prevented by inhibiting protein synthesis in the amygdala, or knocking down ZIF268 by infusing *zif268* antisense oligonucleotides, or by blocking NMDA or β-adrenoceptors. Systemic NMDA or β-adrenoceptor blockade also prevents drug memory reconsolidation. The result is drug memory ‘erasure’ with the consequence that the drug-associated CS can no longer support drug seeking and thereby prevents relapse (see Milton & Everitt, 2010, for review).

The problems associated with chronic drug treatments to prevent relapse elicited by drug-associated CSs and contexts may, however, be circumvented by translating to the clinic our recent understanding of the neural basis of memory reconsolidation, extinction and interactions between these psychological processes. Memory reconsolidation is the process by which brief reactivation of a memory – usually achieved by brief presentations of a reminder CS or context that are insufficient to engage extinction learning – results in the memory becoming destabilized in the brain, a state from which it must be re-stabilized through *de novo* protein synthesis in order to persist (Nader *et al*., 2000b) (Fig. 4). Retrieval-dependent memory plasticity is not a new phenomenon, but experiments by Nader *et al*. (2000a) first demonstrated that conditioned fear memory reconsolidation could be prevented by intra-amygdala protein synthesis inhibition, resulting in memory erasure and subsequent loss of a fear response on subsequent presentation of the CS (Nader *et al*., 2000a,b). We subsequently showed that ZIF268, the protein product of the immediate-early gene *zif268*, was a critical requirement for cued fear memory reconsolidation in the amygdala, and contextual fear memory reconsolidation in the hippocampus (Lee *et al*., 2004).

We then faced a major challenge when seeking to extend the concept of reconsolidation to drug memories that have relevance to addiction because of the marked differences in conditioning history – one or two CS–footshock pairings are used to establish conditioned fear and the fear response of freezing is readily measured. But the self-administration of drugs over many days means that there are many pairings between cocaine and a CS in order for the CS to support drug seeking and precipitate relapse (Lee & Everitt, 2007). It was not clear that such a strongly conditioned CS–cocaine association would undergo reconsolidation at retrieval. However, despite that concern, we went on to show that knockdown of ZIF268 in the amygdala prevented reconsolidation of a CS–cocaine memory and a marked reduction in the ability of the CS subsequently to act as a conditioned reinforcer to maintain high levels of cocaine seeking or to precipitate relapse (Lee *et al*., 2005). Importantly, the CS had been paired with cocaine several hundred times during daily cocaine self-administration sessions, yet relatively small numbers of response-contingent or non-contingent CS presentations at reactivation resulted in memory lability and reconsolidation blockade by ZIF268 antisense oligonucleotide infusions into the BLA at retrieval. In terms of intracellular signalling cascades that may interact with ZIF268 expression, inhibition of protein kinase A has been shown to prevent CS-induced reinstatement of cocaine seeking (Sanchez *et al*., 2010), while inhibition of ERK, and the ERK kinase MEK prevented the reconsolidation of a cocaine-conditioned place preference memory (Miller & Marshall, 2005; Valjent *et al*., 2006).

These data led to the investigation of neurotransmission mechanisms antecedent to the intracellular signalling cascades underlying reconsolidation, both because of their intrinsic importance and the potential for translation to the clinic. A major focus has been on NMDA receptor-mediated glutamate transmission and β-adrenoceptor-mediated noradrenergic signalling, initially as both had been implicated in the memory consolidation process. Numerous studies have shown that an NMDA receptor antagonist or a β-adrenoceptor antagonist given at memory reactivation can result in a loss of the acquired motivational properties of a drug CS and a reduction in cued reinstatement, drug seeking and relapse (Bernardi *et al*., 2006; Lee *et al*., 2006a; Robinson & Franklin, 2007; Fricks-Gleason & Marshall, 2008; Milton *et al*., 2008a,b). These demonstrations of drug memory reconsolidation impairment have been shown across addictive drugs, including cocaine and alcohol, as well as for heroin withdrawal memories (Hellemans *et al*., 2006; Milton & Everitt, 2010, 2012a; Milton, 2013). Generally NMDA receptor antagonism is universally effective in preventing drug memory reconsolidation and there is an established link between NMDA receptor activation and ZIF268 expression in the amygdala at memory retrieval (Milton *et al*., 2008a). There are circumstances, however, in which β-adrenoceptor blockade at reactivation did not prevent reconsolidation, for example an alcohol conditioned place preference (Font & Cunningham, 2012), yet it did decrease responding for an alcohol-associated CS when given in conjunction with alcohol CS memory reactivation in rats having drunk alcohol during daily sessions (Milton *et al*., 2012). There are many conditions yet to be discovered that will either limit or optimize reconsolidation-based treatment interventions, but there is an accumulated body of evidence to support the view that drug memory reconsolidation is a viable target for treatments of addiction (Taylor *et al*., 2009; Milton & Everitt, 2010; Milton, 2013; Torregrossa & Taylor, 2013).

Both NMDA receptor and β-adrenoceptor antagonist treatments given at memory reactivation can not only erase or diminish the impact of pavlovian associations underlying conditioned reinforcement, but also those underlying conditioned approach and PIT (or conditioned motivation), suggesting that the several pavlovian influences on drug seeking and relapse can be diminished by a single treatment given at a CS–drug memory reactivation session (Milton & Everitt, 2010; Milton *et al*., 2010). Here then is a clear advantage of reconsolidation-based treatments for relapse prevention, namely the requirement for only a single or few treatment sessions that could be given in conjunction with currently used cognitive behaviour therapy or even cue exposure treatments. The latter have limited effectiveness in preventing relapse in clinical populations and this is very likely related to the context specificity of extinction learning – extinguishing drug CSs in the clinic does not mean that the cues are extinguished when encountered in the drug user's environment, because the inhibitory CS–no US association formed during extinction learning is expressed predominantly only in the extinction environment (Conklin & Tiffany, 2002). Here there may be another advantage of reconsolidation-based treatments because, so far as is known currently, reconsolidation blockade is not context dependent (Lee & Everitt, 2007). An innovative procedure combining CS–drug memory reconsolidation blockade and enhanced extinction of the drug context using d-cycloserine (DCS) was shown to have greater effectiveness and may have great promise (Torregrossa *et al*., 2010).

The enhancement of drug CS extinction has also been suggested as a means of reducing relapse, encouraged by the ability of DCS to potentiate conditioned fear memory extinction (Davis, 2002; Lee *et al*., 2006b). This attractive possibility has not, however, received much experimental or clinical support (see reviews by Taylor *et al*., 2009; Myers & Carlezon, 2012). Attempts to do so have highlighted a major risk in using DCS to enhance drug CS extinction, which arises through the finding that reconsolidation and extinction are bidirectionally modulated by NMDA receptor antagonists and agonists (Lee *et al*., 2006b). Thus, NMDA receptor blockade prevents fear memory reconsolidation and extinction, but these manipulations have opposite effects on the fear memory – erasing it after reconsolidation blockade and causing the memory to persist if used to prevent extinction. Treatment with DCS does the opposite, enhancing memory persistence if given in conjunction with brief memory reactivation, but enhancing extinction if given in association with repeated CS presentations in an extinction procedure (Lee *et al*., 2006b). Therefore, a critical understanding of the conditions of and limiting variables, often called ‘boundary conditions’, of reconsolidation and extinction is essential if a well-intentioned treatment, say to enhance extinction, is not inadvertently to enhance reconsolidation. In the case of CS–cocaine memories, DCS can certainly potentiate reconsolidation and increase the impact of the CS on drug seeking measured subsequently, but we have not been able to demonstrate DCS enhancement of drug memory extinction (Lee *et al*., 2009). Furthermore, in a preliminary treatment trial in which DCS was used to enhance extinction of a cocaine-associated memory (Price *et al*., 2013), the DCS-treated group of cocaine abusers subsequently showed an increase, not a decrease, in craving elicited by later exposure to the CS. Although not alluded to as an explanation, it is possible that the repeated presentations of the drug CS were insufficient to engage the extinction process and that DCS actually enhanced reconsolidation and strengthened the drug memory, the opposite of the intended outcome. Notwithstanding the conceptual and practical difficulties, the time is perhaps right to consider controlled trials of drug memory reconsolidation blockade in a treatment setting, with the advantage that a clinically available medication could be used (propranolol or other β-adrenoceptor antagonist) in perhaps only one or few treatment sessions (Taylor *et al*., 2009; Milton & Everitt, 2010).

A further and final development to which attention should be drawn is the demonstration that extinction training conducted shortly after a brief memory reactivation, sometimes called ‘extinction in the reconsolidation window’, or ‘super-extinction’ results not only in an extinguished, but a seemingly ‘erased’ memory for the original association (Monfils *et al*., 2009). This was first shown by extinction of a pavlovian fear memory after a brief memory reactivation, which resulted not only in a loss of conditioned fear when tested later, but also a loss of renewal and reinstatement processes that are usually seen after extinction because the original CS–US association is not erased by extinction, only overshadowed by the newly established CS–no US memory. This phenomenon has also been seen in fear conditioning studies in human subjects (Kindt *et al*., 2009; Schiller *et al*., 2010). Thus, it seems that when extinction training follows a brief reactivation to induce memory lability, the original CS–US association is in some sense overwritten by the new CS–no US association, rather than an inhibitory CS–no US memory being formed during extinction that serves to reduce conditioned responding, but only in the same context. However, the retrieval–extinction effect is not always demonstrable and there is much to learn about when the effect is seen and the underlying psychological and neural mechanisms (Hutton-Bedbrook & McNally, 2013). Nevertheless, super-extinction has been shown to disrupt CS–drug memories in rats and human heroin-dependent subjects (Xue *et al*., 2012); a drug conditioned place preference memory and cued relapse to heroin and cocaine seeking could both be super-extinguished by the brief retrieval–extinction training protocol (see Milton & Everitt, 2012b for discussion). Essentially the same procedure was used in a population of in-patients addicted to heroin who were trying to abstain from drug use and the results showed a marked reduction in relapse probability for up to 6 months subsequently (Xue *et al*., 2012). These data require replication, but they indicate that a tried and tested, but clinically sub-optimal, procedure of cue exposure therapy may be made much more effective by the addition of a brief memory reactivation session briefly before extinction training through repeated non-reinforced CS presentations.

## Overview

In summary, this has been a remarkable period of increased understanding of the psychological and neural mechanisms underlying drug reward processes, the taking and seeking of drugs, the neuroadaptations to chronic drug use and the emergence of compulsive drug use and addiction seen in vulnerable individuals. I have in the context of this FENS-EJN award review necessarily highlighted the contributions from our laboratory in Cambridge, but in doing so it was not my intention to diminish or ignore the wealth of data that have been generated by many highly active and successful researchers. The fact is that none of us has the complete answer to addiction, how it develops in some and not the many and why it is so difficult to treat. This probably means there is not an answer, but many, depending on the specific drug and the variety of contexts in which they are taken. However, there are now many clues as to how we might go about introducing new treatments and improving existing ones and that advantage will be taken of these opportunities in the very near future.

## Abbreviations

5CSRTT: five-choice serial reaction-time task
A: action
ADHD: attention deficit hyperactivity disorder
BLA: basolateral amygdala
CeN: central amygdala
CS: conditioned stimulus
D3R: D3 receptor
DA: dopamine
DCS: d-cycloserine
DLS: dosolateral striatum
DMS: dorsomedial striatum
ERK: extracellular signal-regulated kinase
FR: fixed ratio
HR: high responder
LI: low impulsivity
NAC: *N*-acetyl cysteine
NAcb: nucleus accumbens
NMDA: *N*-methyl-d-aspartate
O: outcome
OCD: obsessive-compulsive disorder
OFC: orbital prefrontal cortex
PFC: prefrontal cortex
PIT: pavlovian-instrumental transfer
SIP: schedule-induced polydipsia
S-R: stimulus–response
SSRI: serotonin-selective reuptake inhibitor
